# Factors associated with late Human Immunodeficiency Virus (HIV) diagnosis among peoples living with it, Northwest Ethiopia: hospital based unmatched case-control study

**DOI:** 10.1186/s12889-016-3727-0

**Published:** 2016-10-12

**Authors:** Abebayehu Bitew Aniley, Tadesse Awoke Ayele, Ejigu Gebeye Zeleke, Assefa Andargie Kassa

**Affiliations:** 1 Department of Public Health, College of Medicine and Health Sciences, Wollo University, P.O. Box: 1145, Dessiee, Ethiopia; 2 Department of Epidemiology and Biostatistics, Institute of Public Health, College of Medicine and Health Sciences, University of Gondar, P.O. Box:196, Gondar, Ethiopia

**Keywords:** Late HIV diagnosis, Associated factors, Unmatched case-control, Ethiopia

## Abstract

**Background:**

Early HIV diagnosis and access to treatment is one of the most effective ways to prevent its further spread and to protect the health of those living with the virus. However, delay in diagnosis is the major risk factor for uptake of and response to antiretroviral therapy.

**Methods:**

Institution-based unmatched case-control study design was used in the study. The study was conducted in Debre-Markos and Finote-Selam Hospitals, Northwest Ethiopia. Cases were people living with HIV who had CD4 count <350cells/mm^3^ or WHO clinical stage III and IV regardless of the CD4 count at first presentation and controls were those who had CD4 count ≥350cells/mm^3^ or WHO clinical stage I and II. If both criteria were available, the CD4 count was used in the study as World Health Organization recommended. A total of 392 respondents (196 cases and 196 controls) were recruited and selected systematically. The data were collected by trained nurses using chart review and interviewer administered structured questionnaire. Binary Logistic Regression Model was used to identify the factors associated with late HIV diagnosis.

**Results:**

About 95.9 % of study participants provided complete response. Having no understanding, compared to having understanding, about HIV/AIDS (AOR = 1.7, 95 %CI = 1.08–2.79) and ART (AOR = 2.1, 95 %CI: 1.25–3.72), being tested as a result of symptoms/ illness, compared to being tested for risk exposure (inverted AOR =2.5, 95 %CI: 1.64–4.76), and acquiring HIV through sexual contact, compared to acquiring it through other modes (AOR = 2.5, 95 %CI = 1.52–4.76) were positively and independently associated with late HIV diagnosis.

**Conclusions:**

Unlike perceived HIV stigma, having no understanding about HIV and ART, being tested for presence of symptoms/illness, and acquiring HIV through sexual contact were independent and significant factors for late HIV diagnosis.

## Background

For over 20 years, human immunodeficiency virus (HIV) infection and acquired immunodeficiency syndrome (AIDS) have been significant public health problems particularly in low- and middle- income countries, with two-thirds of the world’s HIV-infected population living in Africa [1]. It is also one of the most serious public health and development challenges in Ethiopia [2].

Acquired Immune Deficiency Syndrome (AIDS) is also one of the most serious public health and development challenges in Ethiopia. It is affecting all sectors of Ethiopian society. According to the 2011 EDHS, 1.5 % of adults age 15–49 were infected with HIV and heterosexual contact accounts for the great majority of HIV transmission in the country. The future course of its epidemic in Ethiopia depends on a number of factors including HIV/AIDS-related knowledge, social stigmatization, risk behavior modification, access to high-quality services for sexually transmitted infections (STIs), provision and uptake of HIV counseling and testing, and access to antiretroviral therapy (ART) [2].

Early diagnosis and access to treatment is one of the most effective ways to prevent the further spread of HIV and to protect the health of those living with the virus [3]. It helps people with HIV to timely get and appropriately use antiretroviral treatment. The timely and appropriate use of antiretroviral treatment lowers the amount of virus in their body and can dramatically reduce their risk of morbidity, mortality and it has been conclusively shown to decrease HIV transmission by greater than 90 % [4–6].

There is quicker and easier HIV testing, and more people have been tested and know more about how to prevent HIV infection today than at any other time in the history of the disease. However, the presence of too few people with HIV who are aware of their infection remains being one of the significant challenges of infection prevention strategies. Because, the majorities of people with HIV who do not know they are infected are significantly transmitting HIV and contribute half of the new infections [3]. Delay in knowing the HIV positive status also results the advancement of the disease which poses a less favorable clinical course, with reduced or incomplete treatment response, more rapid clinical progression, and higher risk of mortality on the victims. In addition, it generates a considerable and avoidable resource burden to the healthcare system [5].

To enhance earlier diagnosis and timely presentation for care in Ethiopia, HIV counseling and testing services are integrated into existing health and social welfare services. Providers initiated HIV testing and counseling is also integrated as part of standard clinical management and care in all health facilities [7]. These services also help people living with HIV to present at earlier stages of their illness and access HIV/AIDS services timely and improve their health status. Those starting treatment early are found to have improved survival [8].

However, even if there is quicker and easier HIV testing, and more people have been tested and know more about how to prevent HIV infection today than at any other time in the history of the disease, the presence of too few people with HIV who are aware of their infection remains being one of the significant challenges of infection prevention strategies. Because, the majorities of people with HIV who do not know they are infected are significantly transmitting HIV and contribute half of the new infections [3]. Delay in knowing the HIV positive status also results late diagnosis and the advancement of the disease which poses a less favorable clinical course, with reduced or incomplete treatment response, more rapid clinical progression, and higher risk of mortality on the victims. In addition, it generates a considerable and avoidable resource burden to the healthcare system [6].

Many factors are found to keep people from finding out their HIV status and determine the future course of its epidemic. These include socio-demographic factors like older age, male gender, living in rural areas [5–10] and behavioral factors like HIV/AIDS and ART-related knowledge, attitude and belief, social stigma, risk behavior modification, access to high-quality services for sexually transmitted infections (STIs), provision and uptake of HIV counseling and testing, and access to antiretroviral therapy (ART) [11–20].

## Methods

Unmatched case-control study design was conducted from April 15 to March 20, 2013 in Debre-Markos and Finote-Selam Hospitals, Northwest Ethiopia. Debre-Markos and Finote-Selam are the capitals of East Gojjam and West Gojjam Zones in Amhara Regional state, respectively. Debre-Markos is found 300 kilometers northwest of the capital of the Country, Addis Ababa and 265 kilometers Southeast of Bahir Dar, the capital of Amhara Regional state. Similarly, Finote-Selam is located 387 kilometers northwest of Addis Ababa and 166 kilometers southeast of Bahir Dar. The two hospitals provide voluntary counseling and testing (VCT), prevention of mother to child transmission (PMTCT), antiretroviral therapy (ART) and treatment of opportunistic infection services. The study population were cases and controls of people with HIV, age ≥ 18 years, who visited the ART clinics of the two hospitals during the data collection period. Cases were individuals who had cluster of differentiation 4 (CD4) cell count ˂350cells/mm^3^ or those who had WHO clinical stage III or IV at their presentation during first HIV diagnosis. Controls were individuals who had CD4 cell count ≥350cells/mm^3^ and those who had WHO clinical stage I or II at their presentation during first HIV diagnosis. Where clinical and immunological classifications were both available, immune status, reflected by CD4 count, was preferred as recommended by WHO [21–23].

The sample size was calculated using Epi info version 7 developed by Communicable Disease Control after considering the following assumptions: proportion of high perceived HIV stigma, a factor which provided large sample size, were 9.4 and 22.5 % among controls and cases respectively, 95 % CI, 90 % power, 1 to 1 case to control ratio and 10 % non-response rate. The total sample size was 392 (196 cases and 196 controls [14]). Cases and controls were recruited using systematic random sampling technique with a sampling interval (k) of 2 after the total sample size was allocated proportionally to the Hospitals based on the ART clients load. Therefore, 262 respondents (131 cases and 131 controls) were recruited from Debre-Markos Hospital and the remaining 130 respondents (65 cases and 65 controls) were selected from Finote-Selam Hospital.

The data was collected by trained nurses working in the Hospitals after The data collection tool was pre-coded, pretested and interview-based structured questionnaire developed after reviewing relevant literatures on the issue.

Comprehensive HIV knowledge was determined based on 5 items which were used in Ethiopian Demographic and Health Survey (EDHS) 2011, which is part of the worldwide measure of Demographic and Health Survey project funded by the United States Agency for International Development (USAID) and implemented by the Ethiopian Central Statistical Agency. An individual who correctly answered all of the items was considered as having comprehensive HIV knowledge, but if s/he failed to answer at least 1 of them, s/he was considered as s/he didn’t’ have it [2]. Similarly, attitude of respondents, towards people with HIV/AIDS was measured using 4 “yes/no” attitude questions used by EDHS. One point was given for “yes” responses and zero point for “no” responses. The total score of a respondent, therefore, ranged from 0 to 4. Scores of individuals less than the mean score of the study population was categorized as having “negative” attitude and scores greater than or equal to the mean score represented “positive” attitude [2]. Understanding about HIV/AIDS was also determined by 5 “yes/no” HIV/AIDS questions adopted from related literatures. “Yes” responses, given 1 point, indicated rejecting the common truth about HIV/AIDS, while “no” response, given 0 point, indicated accepting it. Based on a similar technique used in the determination of attitude about HIV/AIDS, it was categorized as “good” or “poor”. In addition, perceived HIV stigma, categorized as “high” or “low”, was measured using 14 “yes/no” items which were validated and recommended in different studies. These items were related to participants’ perceptions, about how their partners, friends, families and the community would react to them if their HIV positive status was known [24–26]. Further, knowledge about ART was measured using 6 “yes/no” items and labeled as “good” or “poor” based on the total score. The level of understanding about ART was also determined using 6 “yes/no” items and categorized as “have no understanding” and “have understanding” using similar techniques.

To maintain the quality of data obtained, the questionnaire was translated to “Amharic”, the local language, and back to English language to check its consistency by individuals who had related experience. In addition, the questionnaire was pre-tested and there was regular supervision and timely check-up of the completeness and consistency of responses to questions throughout the data collection period. Written informed consent from each respondent was obtained and ethical approval was received from the Ethical Review Board of University of Gondar and permission letter was also received from the Hospital Administrators.

The data were entered into a pre-designed format in Epi Ifo version 3.5.1 developed by Communicable Disease Control and transferred to SPSS version 16 for analysis. Binary logistic regression analysis was applied to identify the independent and significant factors. Before running the regression analysis, assumptions to apply binary logistic regression were checked and satisfied. These include ratio of cases to independent variables (greater than 10 to 1), overall relationship of independent variables with the dependent variable (Model chi-square, *p*-value < 0.05), Hosmer and Lemshow test of model goodness of fit (*p*-value > 0.05) and the standard errors of model coefficients were less than 2.

Description of the main analysis findings was done using frequencies, percentages and summary statistics. To identify the factors associated with late HIV diagnosis, first, uni-variate analysis, in the binary logistic regression, between each independent variable and the dependant one was carried out. Those variables with a *p*-value ≤0.2 were then included in the multiple analysis of binary logistic regression to decrease the effect of residual confounding. In the multiple analysis, the Backward Likelihood Ratio Method was used to identify the independent and significant factors at 0.05 level of significance. Odds ratio and 95 % confidence interval were used in the interpretation of the result.

## Results

### Socio-demographic characteristics

Out of the total of 392 people living with HIV (196 cases and 196 controls), 376 (96 % response rate) provided appropriate data. Out of these, 135 (35.9 %) were males and 241 (64.1 %) were females and 187 (49.7 %) were cases and 189 (50.3 %) were controls. Among the cases, 123 (65.8 %) were from Debre-Markos and 64 (34.2 %) were from Finote-Selam Hospitals. Similarly, among the controls, 125 (66.1 %) and 64 (33.9 %) were from Debre-Markos and Finote-Selam Hospitals respectively.

From the total 187 cases and 189 controls, nearly half (100 (53.5 %)) of the cases and about two-third (129 (68.3 %)) of the controls were tested positive after the year 2008. One hundred twenty one (64.7 %) of cases and 120 (63.5 %) of controls were females. The median age for both cases and controls was 30 years with a respective interquartile range (IQR) of [27–38] and [25–36] years. The result also showed that 157 (84.0 %) of cases and 156 (82.5 %) of controls live in urban areas. Marital status composition of participants showed that 87 (46.5 %) of cases and 100 (52.9 %) of controls were married. Forty five (24.1 %) of cases and 57 (30.2 %) of controls couldn’t read/write. Regarding occupational status, 30 (16 .0 %) of cases and 43 (22.8 %) of controls had no job, 24 (12.8 %) of cases and 29 (15.3 %) of controls were farmers, 42 (22.5 %) of cases and 40 (21.2 %) of controls were government employed, and others category includes those working in private companies or non-governmental organizations. In addition, 83 (44.4 %) of cases and 94 (49.7 %) of controls were living with their spouse and 2 (1.1 %) of both cases and controls were living with other people which were not their blood relatives. About 99 (52.9 %) of cases and 90 (47.6 %) of controls were living in a rented house (Table 1).

**Table 1:** Socio demographic characteristics of respondents in Debre-Markos and Finote-Selam Hospitals, May 2013

Characteristics	Cases N (%)	Controls N (%)	Total N (%)
Year of positive diagnosis			
Before 2007	30 (16.0)	15 (7.9)	45 (12.0)
2007–2008	57 (30.5)	45 (23.8)	102 (27.1)
After 2008	100 (53.5)	129 (68.3)	229 (60.9)
Sex			
Male	66 (35.3)	69 (36.5)	135 (35.9)
Female	121 (64.7)	120 (63.5)	241 (64.1)
Pregnancy status			
Pregnant	16 (13.2)	11 (9.2)	27 (11.2)
Not pregnant	105 (86.2)	109 (90.8)	214 (88.8)
Residence			
Urban	157 (84.0)	156 (82.5)	313 (83.2)
Rural	30 (16.0)	33 (17.5)	63 (16.8)
Marital status			
Single	35 (18.7)	29 (15.3)	64 (17.0)
Married	87 (46.5)	100 (52.9)	187 (49.7)
Divorced/separated	40 (21.4)	38 (20.1)	78 (20.7)
Widowed	25 (13.4)	22 (11.6)	47 (12.5)
Educational status			
Cannot read/write	45 (24.1)	57 (30.2)	102 (27.1)
Read/write only	23 (12.3)	15 (7.9)	38 (10.1)
Primary	42 (22.5)	40 (21.2)	82 (21.8)
Secondary	53 (28.3)	45 (23.8)	98 (26.1)
Tertiary (college/university)	24 (12.8)	32 (16.9)	56 (14.9)
Occupational status			
No job	30 (16.0)	43 (22.8)	73 (19.4)
Farmer	24 (12.8)	29 (15.3)	53 (14.1)
Government employee	42 (22.5)	40 (21.2)	82 (21.8)
Merchant	45 (24.1)	30 (15.9)	75 (19.9)
Daily laborer	25 (13.4)	27 (14.3)	52 (13.8)
Student	4 (2.1)	4 (2.1)	8 (2.1)
Commercial sex worker	7 (3.7)	8 (4.2)	15 (4.0)
Driver	6 (3.2)	1 (0.5)	7 (1.9)
Others	4 (2.1)	7 (3.7)	11 (2.9)
Living arrangement			
Alone	49 (26.2)	44 (23.3)	93 (24.7)
With family	53 (28.3)	49 (25.9)	102 (27.1)
With spouse	83 (44.4)	94 (49.7)	177 (47.1)
Others	2 (1.1)	2 (1.1)	4 (1.1)
Ownership of living house			
Own	88 (47.1)	99 (52.4)	187 (49.7)
Rented	99 (52.9)	90 (47.6)	1890.3)

Among the total of 376 participants, 158 (84.5 %) of cases and 168 (88.9 %) of controls had ever heard about HIV/AIDS before their first HIV positive diagnosis. Only 32 (20.3 %) of cases and 58 (34.5 %) of controls had comprehensive HIV knowledge.

Similarly, the result for the total attitude score from each indicator showed that about one-third (105 (66.5 %)) of cases and nearly half (82 (48.8 %)) of controls had “negative” attitude towards people living with HIV.

The cumulative belief score result displayed that about 115 (72.8 %) of cases and 94 (56.0 %) of controls had “poor” belief about HIV/AIDS.

From those respondents who had ever heard about HIV/AIDS before their HIV positive diagnosis, 60 (38 %) of cases and 73 (43.5 %) of controls perceived that they might have HIV before knowing their HIV positive status. About two-third (69.0 %) of cases and nearly half (48.7 %) of controls reported that their main reason to be tested for HIV was the presence of symptoms or illness. One hundred one (86.1 %) of cases and 133 (70.4 %) of controls thought as they had acquired HIV through sexual contact. In addition, 3 (1.6 %) of cases and 7 (3.7 %) of controls had reported that they acquired HIV by other mechanism including during genital mutilation and wound contact.

More than half of both cases and controls, who ever heard about HIVAIDS, reported that they would feel as they were guilty if their HIV test result was positive. In addition, if other people knew they had HIV, more than half of both cases and controls perceived that they would be stigmatized by their friends, families and the society. The total score result from the 14 HIV stigma items showed that 117 (74.1 %) of cases and 91 (54.2 %) of controls had “high” perceived stigma.

One hundred thirty four (71.7 %) of cases and 116 (61.4 %) of controls had ever received treatment from medical clinic. Twenty five (13.4 %) of cases and 20 (10.6 %) of controls were ever treated by traditional healer/s. In addition, 132 (70.6 %) of cases and 102 (54.0 %) of controls had opportunistic illness during their first HIV positive diagnosis. Fifty two (27.8 %) of cases and 43 (22.8 %) of controls reported that they had STI during or before the diagnosis.

Among the respondents who had ever heard about HIV/AIDS, 145 (91.8 %) of cases and 155 (92.3 %) of controls had also heard about ART. The total ART knowledge score based on 6 indicators indicated that 71 (44.9 %) of cases and 58 (34.5 %) of controls had “poor” ART knowledge.

Similarly, the total ART belief score on the 4 indicators showed that 106 (73.1 %) of cases and 85 (54.8 %) of controls had “poor” belief about ART.

### Factors associated with late HIV diagnosis

Factors which were associated with late HIV diagnosis in the univariate logistic regression at a 0.2 level of significant include age, presence of comprehensive HIV knowledge, attitude towards people living with HIV/AIDS, understanding about HIV/AIDS, perceived HIV stigma, knowledge about ART, understanding about ART, number of sexual partners before HIV positive diagnosis, main reason for wanting to test, mode of HIV acquisition, time of HIV negative test result before the positive diagnosis, receiving treatment from medical clinic, receiving treatment from pharmacy/drug store, presence of hospital admission, presence of opportunistic illness, time of latest HIV negative test result, year of HIV positive diagnosis, and distance of the nearest healthcare facility. However, religion and marital status were not significantly associated with late HIV diagnosis.

These factors were included in the multiple logistic regression analysis. Variables which were significantly associated with Late HIV Diagnosis at a 0.05 level of significance in the multiple logistic regression analysis were belief about HIV/AIDS, belief about ART, main reason for wanting to test, mode of HIV acquisition and year of HIV positive diagnosis. However, the primary hypothesized factor, perceived HIV stigma, was marginally significant.

Respondents who had no understanding about HIV/AIDS were about 1.7 times more likely to be diagnosed late as compared to those who had understanding (AOR = 1.7, 95 % CI = 1.08–2.79). Similarly, individuals who had no understanding about ART were about 2 times at risk of late HIV diagnosis as compared to those who had understanding (AOR = 2.1, 95 % CI = 1.25–3.72) (Table 2).

**Table 2:** Factors associated with Late HIV Diagnosis in Debre-Markos and Finote-Selam hospitals, Northwest Ethiopia, April 2013

Characteristics	Cases N	Controls N	COR (95 %CI)	AOR (95 %CI)	*P*-value
Comprehensive HIV knowledge^c^					0.566
Had	32	58	1^a^	1^a^	
Didn’t have	126	110	2.1 (1.26–3.43)	1.2(0.64–2.29)	
Attitude about HIV^c^					
Positive	53	86	1^a^	1^a^	0.310
Negative	105	82	2.1(1.33–3.25)	1.3 (0.76–2.39)	
Understanding about HIV/AIDS					
Had	43	74	1^a^	1^a^	<0.001
Had no	115	94	2.1 (1.32–3.349)	1.7 (1.08–2.79)	
Perceived HIV stigma					
Low	41	77	1^a^		0.200
High	117	91	2.4 (1.51–3.85)	1.7 (1–2.89)^b^	
ART knowledge^c^					
Good	87	110	1^a^	1^a^	0.775
Poor	71	58	1.5 (1–2.42)^b^	1.1 (0.61–1.93)	
Understanding about ART					
Had	39	70	1^a^	1^a^	0.015
Had no	106	85	2.2 (1.38–3.63)	2.1 (1.25–3.72)	
Reason to test					
Symptoms	129	92	1^a^	1^a^	0.003
Screening	22	35	0.4 (0.25–0.81)	0.5 (0.27–1.06)^b^	
Risk exposure	36	62	0.4 (0.25–0.68)	0.4 (0.21–0.61)	
Mode of HIV acquisition					
Sexual contact	161	133	1^a^	1^a^	0.008
Other methods	26	56	0.4 (0.23–0.64)	0.4 (0.21–0.66)	
Opportunistic illness^c^					
Absent	55	87	1^a^	1^a^	0.604
Present	132	102	2.0 (1.34–3.13)	0.9 (0.52–1.48)	
Year of diagnosis					
Before 2007	30	15	1^a^	1^a^	0.014
2007–2008	57	45	0.6 (0.3–1.32)	0.6 (0.3–1.42)	
After 2008	100	129	0.4 (0.2–0.76)	0.3 (0.17–0.72)	

*N* number of respondents, *COR* crude odds ratio, *CI* confidence interval, *AOR* adjusted odds ratio

^a^Reference category, ^b^mariginally significant at 0.05, ^c^variables which were not significantly associated with late HIV diagnosis in the multiple analysis

In addition, study participants who were tested as a result of HIV risk exposure had 60 % decreased risk of being diagnosed late as compared to those tested due to presence of symptoms/illness (AOR = 0.4, 95 % CI: 0.21–0.61). Even though it was marginally significant, participants tested for screening purpose, as compared to those tested due to presence of symptoms/illness, had 50 % decreased risk of being diagnosed late (AOR = 0.5, 95 % CI: 0.27–1.06).

As compared to those who acquired HIV through sexual contact, respondents who acquired HIV through other modes of transmission including sharp materials, blood transfusion, intravenous drug use and wound contact had 50 % decreased risk of being diagnosed late (AOR = 0.4, 95 %CI = 0.21–0.66).

Study participants who were tested after 2008 had 70 % decreased risk of late HIV diagnosis as compared to individuals who were tested before 2007 (AOR = 0.3, 95 %CI = 0.17–0.72). However, there was no significant difference between those diagnosed before 2007 and 2007–2008 (AOR = 0.6, 95 %CI = 0.3–1.42).

## Discussion

Diagnosing HIV at its earlier stage has easily recognizable importance for those living with it to timely link with and get maximal benefit from HIV care and treatment service. It also helps to strengthen actions taken to prevent transmission of the infection particularly from those who are unaware of their infection. However, different factors are identified to hinder early HIV diagnosis.

The primary hypothesized factor for late HIV diagnosis in this study was perceived HIV stigma. It was marginally significant in the multiple logistic regression analysis result. Respondents who had high perceived HIV stigma, as compared to those who had low perceived HIV stigma, were 1.7 times more likely to be diagnosed (AOR = 1.7, 95 % CI: 1–2.89), which is similar with the result of a case-control study in South Wollo, Northeast Ethiopia [14]. Findings evidenced that there was consistent improvement in the immune status of people living with HIV at their first diagnosis across time trends because of increased knowledge about HIV/AIDS and access to HIV testing and counseling services [9, 27].

In addition, the earlier study considered individuals who were greater or equal to 15 years old; while this study included individuals greater or equal to 18 years old. Individuals with age 15–17 years old are usually considered as they are immature by the society and they might feel that their parents, families and the society would stigmatize them due to their HIV acquisition in their earlier age. This, as a result, might increase perceived HIV stigma.

Study participants who had no understanding about HIV/AIDS were more likely to be diagnosed late at first presentation as compared to those who had understanding. This could be due to lack of comprehensive HIV knowledge, which was significant predictor of late HIV diagnosis in the univariate analysis, even if it was not in the multivariate one. Because, individuals who had no understanding about HIV/AIDS, compared to those having understanding, had no comprehensive HIV knowledge (COR = 2.6, 95 %CI = 1.57–4.27). A cases-case comparison study conducted in Venezuela supported this possible explanation which identified low knowledge of HIV/AIDS being the main barriers to HIV testing [13].

Similarly, participants who had poor belief about ART were more likely to be diagnosed late as compared to those who had good ART belief, which is strengthened by a case-control study finding in South Wollo that identified believing ART has side effects increased late presentation to HIV care.

In addition, individuals who were tested due to the presence of symptoms or illness were more likely to be diagnosed late for HIV as compared to those tested for both HIV risk exposure and screening purpose. This could be due to the difference in the time interval between infection and diagnosis. Because, presence of symptoms or illness usually come in the later stage of HIV infection. This result is consistent with a finding in New Zealand [18] and South Korea [11].

Sexual contact, as compared to other modes of HIV transmission, was also found to be another risk factor for late HIV diagnosis at first presentation. The possible justification for this could be the high proportion of people living with HIV who acquired it through sexual contact, perceiving that they had HIV as a result of their immoral act and fear of reactions from other people.

On the other hand, those who acquired HIV via other modes of HIV transmission might not believe that they contracted due to their sin. This finding is similar with study results from India [10] and Brussels and France [7].

### Strengths and limitations of the study

Despite many studies on HIV related problems, there was no study conducted on factors associated with late HIV diagnosis in Ethiopia before. Therefore, this study would probably be the first on this specific issue. It also tried to identify possible factors through reviewing related literatures.

Since this study was based on a case-control design, there would be recall bias that might affect the actual result. All the study participants were also selected without specification of their year of first HIV positive diagnosis which could increase the recall bias.

## Conclusion

The primary hypothesized factor, perceived HIV stigma, was not independent and significant predictor of late HIV diagnosis. However, having poor belief about HIV/AIDS and ART, wanting to test as a result of presence of symptoms or illness, and acquiring HIV through sexual contact were independent and significant factors associated with late HIV diagnosis among people living with HIV in Debre-Markos and Finote-Selam hospitals.

## Abbreviations

AIDS: Acquired Immuno-Deficiency Syndrome
ART: Anti-Retroviral Treatment
CDC: Center for Disease Control and prevention
EDHS: Ethiopian Demographic and Health Survey
HCT: HIV Counseling and Testing
HIV: Human Immunodeficiency Virus
IQR: Inter Quartile Range
SPSS: Software Package for Social Sciences
STIs: Sexually Transmitted Infections
USAID: United States Agency for International Development
VCT: Voluntary Counseling and Testing
WHO: World Health Organization
